# T-type calcium channel modulation by hydrogen sulfide in neuropathic pain conditions

**DOI:** 10.3389/fphar.2023.1212800

**Published:** 2023-07-17

**Authors:** Maricruz Rangel-Galván, Violeta Rangel-Galván, Alejandro Rangel-Huerta

**Affiliations:** ^1^ Biothecnology Department, Metropolitan Polytechnic University of Puebla, Puebla, Puebla, Mexico; ^2^ Nursing and Physiotherapy Department, University of Professional Development, Tijuana, Baja California, Mexico; ^3^ Faculty of Computer Science, Meritorious Autonomous University of Puebla, Puebla, Puebla, Mexico

**Keywords:** T-type calcium channels, Cav3.2, hydrogen sulfide, gasotransmitters, neuropathic pain

## Abstract

Neuropathic pain can appear as a direct or indirect nerve damage lesion or disease that affects the somatosensory nervous system. If the neurons are damaged or indirectly stimulated, immune cells contribute significantly to inflammatory and neuropathic pain. After nerve injury, peripheral macrophages/spinal microglia accumulate around damaged neurons, producing endogenous hydrogen sulfide (H_2_S) through the cystathionine-γ-lyase (CSE) enzyme. H_2_S has a pronociceptive modulation on the Ca_v_3.2 subtype, the predominant Ca_v_3 isoform involved in pain processes. The present review provides relevant information about H_2_S modulation on the Ca_v_3.2 T-type channels in neuropathic pain conditions. We have discussed that the dual effect of H_2_S on T-type channels is concentration-dependent, that is, an inhibitory effect is seen at low concentrations of 10 µM and an augmentation effect on T-current at 100 µM. The modulation mechanism of the Ca_v_3.2 channel by H_2_S involves the direct participation of the redox/Zn^2+^ affinity site located in the His191 in the extracellular loop of domain I of the channel, involving a group of extracellular cysteines, comprising C114, C123, C128, and C1333, that can modify the local redox environment. The indirect interaction pathways involve the regulation of the Ca_v_3.2 channel through cytokines, kinases, and post-translational regulators of channel expression. The findings conclude that the CSE/H_2_S/Ca_v_3.2 pathway could be a promising therapeutic target for neuropathic pain disorders.

## 1 Introduction

The prevalence of neuropathic pain is currently estimated to be approximately 7%–10% of the global population. The International Association for the Study of Pain (IASP) states that neuropathic pain is caused by injury or disease in the somatosensory system. In fact, neuropathic pain is caused by both primary injuries and collateral damage due to different chronic diseases. The most common diseases include diabetic peripheral neuropathy, trigeminal neuralgia, and postherpetic neuralgia (Zilliox, 2017; Finnerup et al., 2021). Most neuropathic pain symptoms are associated with disturbances in sensitization mechanisms and excessive atypical neuronal activity. These symptoms are allodynia, a pain sensation due to normally innocuous stimulation, and hyperalgesia, abnormally increased pain sensitivity. Furthermore, an abnormal increase in signaling responses has been observed along three different conduction fibers, namely, Aβ, Aδ, and C fibers, which are the primary afferent nociceptive pathways of the somatosensory system (Colloca et al., 2017; Finnerup et al., 2021). Some conventional pharmacological therapies for treating neuropathic pain include tricyclic antidepressants, serotonin–noradrenaline reuptake inhibitors, and gabapentin and pregabalin anticonvulsant compounds, which are first-line drugs. Second-line and third-line drugs include lidocaine and opioids, respectively. Because these treatments provide only symptomatic relief with a usually late effect period of 2–4 weeks, modest efficacy (∼50% patient pain reduction), and various secondary side effects, research is still being performed to develop new treatment methods to cure neuropathic pain conditions. Successful treatment needs an understanding of the mechanism of action of this disease (Gilron et al., 2015; Gierthmühlen and Baron, 2016; Fornasari, 2017).

**FIGURE 1:**
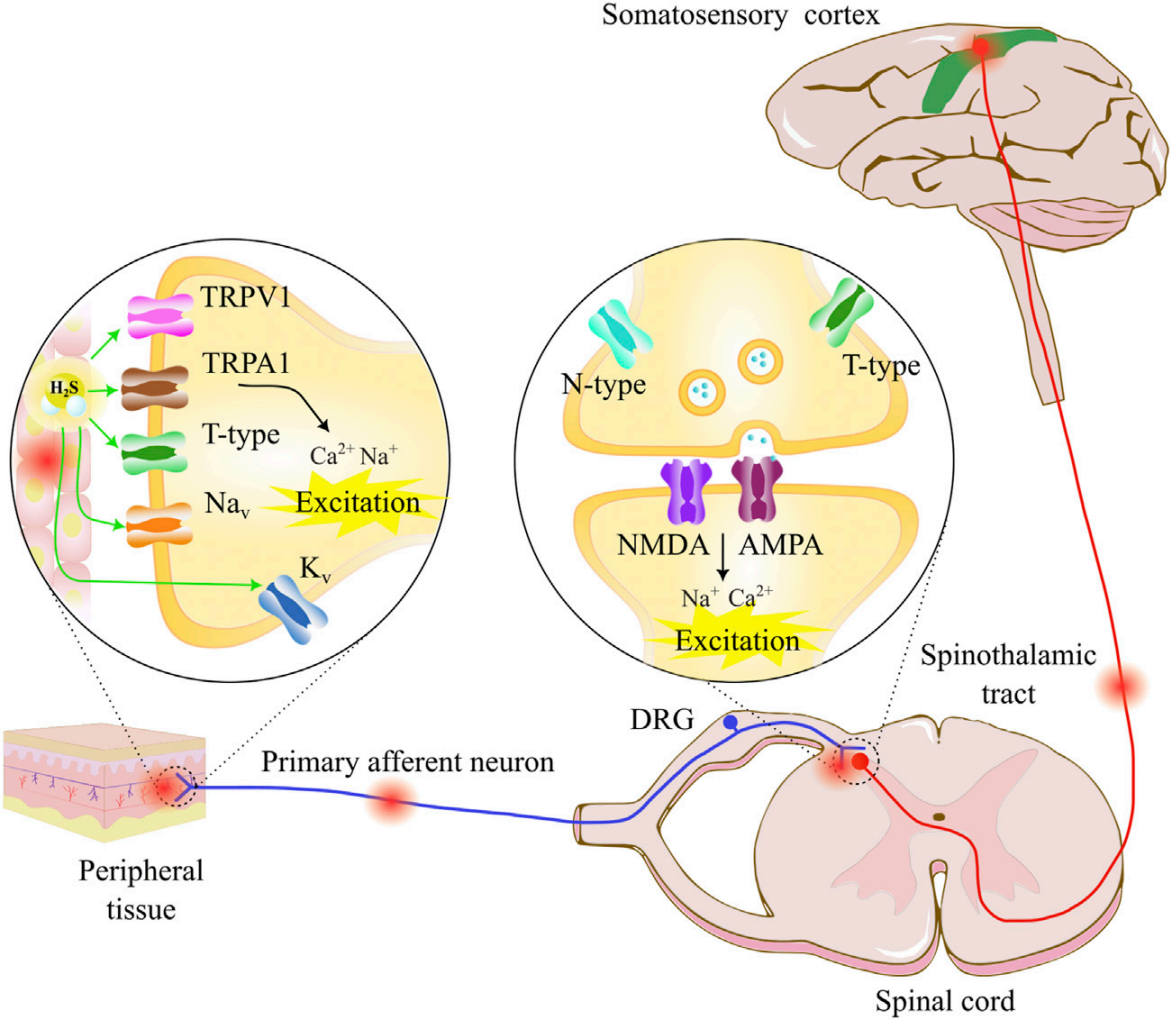
Pain transmission starts with a noxious stimulus in the peripheral tissue, which is detected by various types of nociceptive receptors, such as TRPV for heat perception and TRPA for cold sensation. T-type calcium channels, voltage-gated sodium channels (Na_v_s), and voltage-gated potassium channels (K_v_s) participate in nociceptive neural transmission. The nociceptive information travels through the primary afferent neurons to the dorsal horn neurons, where presynaptic N-type and T-type calcium channels participate in neurotransmitter release, and the postsynaptic glutamate receptors AMPA and NMDA transmit pain sensation through the spinothalamic tract to the thalamus and the somatosensory cortex. Neuropathic pain can appear as a direct or indirect nerve damage, lesion, or disease that affects the somatosensory nervous system (periphery and/or central part). The pain sensation can appear as a response to noxious or non-noxious stimuli. The molecular mechanism involves a hyperexcitation of the somatosensory neurons through ionic channel dysfunction expressed in the neurons. The hydrogen sulfide (H_2_S) modulator in the pathway can interact with various ion channels, where the H_2_S/T-type calcium channel pathway represents an important pharmacological target.

The discussion remains about the origins of neuropathic pain symptoms, emphasizing aspects related to ion channel signaling alterations distributed along the afferent neurons (Colloca et al., 2017). To understand the underlying mechanisms of neuropathic pain, it is necessary to know some essential aspects of the nociceptive sensory pathway (Figure 1). The first contact with external stimuli occurs in the peripheral nociceptive terminals. Signaling initiation is a transduction of stimuli due to changes in membrane potential in peripheral afferents. Their connections continue in the dorsal horn of the spinal cord which in turn transmits this sensory information to the somatosensory cortex through the spinothalamic tract projection neurons (Yam et al., 2018). Several types of ion channels are recruited into the signaling pain pathway, such as the transient receptor potential (TRP) family, acid-sensing ion channel (ASIC), voltage-gated sodium channels (Na_v_s), voltage-gated potassium channels (K_v_s), voltage-gated calcium channels (Ca_v_s), and hyperpolarization-activated cyclic nucleotide-gated channels (HCNs) (Finnerup et al., 2021). Abnormal signaling of pain pathways in the somatosensory system results in neuropathic pain associated with an excitatory or inhibitory imbalance of the electrophysiological responses of neural membrane ion channels. Indeed, in the neuropathic pain signaling pathway, a chronic injury or disease alters the functions of different ion channels expressed in the neural membrane (Gilron et al., 2015; Gierthmühlen and Baron, 2016).

Voltage-gated calcium channels are widely distributed in the central nervous system (CNS) and the peripheral nervous system (PNS) regions, regulating neurotransmitter release, establishing excitability, and participating in cellular neurotransmission in somatosensory nociceptive pathways. They also regulate the rhythmicity of neural activity and initiate other metabolic pathways, such as releasing neuromodulators and gasotransmitters. Afferent neurons of the primary sensory system detect and respond to local damage with enhanced signaling as a response to noxious stimuli. These nociceptors recruit sodium channels (Na_v_1.7, Na_v_1.8, and Na_v_1.9) and calcium channels (Ca_v_2.2 N-type and Ca_v_3.2 T-type) essential for transduction and transmission signaling in nociceptive pain (Colloca et al., 2017; Fornasari, 2017; Ghazisaeidi et al., 2023). The N-type and T-type voltage-gated calcium channels contribute to nociceptive transmission by modulating the release of pain mediators, such as glutamate and substance P, over the dorsal horn of the spinal cord. It has been demonstrated that these channels upregulate their function and expression after nerve injury, leading to pathological conditions. In addition, it has been confirmed that targeting Ca_v_3.2 T-type channels can avoid hypersensitivity to pain associated with chemotherapy-induced peripheral neuropathy (CIPN) and diabetic neuropathy (Ghazisaeidi et al., 2023). Therefore, the findings conclude that calcium channels are important drug targets for various neuronal disorders, including neuropathic pain. Indeed, calcium channels of the Ca_v_3.2 subtype are widely expressed in neurons of the dorsal root ganglion and the spinal cord, increasing their population in conditions of injury and neural damage (Finnerup et al., 2021).

Neuropathic pain indicates direct or indirect damage to the neural signaling pathway, while inflammatory pain is caused by inflammatory mediators such as cytokines released by immune cells. If the afferents are damaged or indirectly stimulated, immune cells contribute significantly to inflammatory and neuropathic pain. In this case, peripheral macrophages or spinal microglia accumulate by proliferation, infiltration, or migration around peripheral neurons. These cells release proinflammatory and/or pronociceptive mediators that act on the nociceptive pathway to produce peripheral sensitization (Domoto et al., 2021). In primary afferent neurons, macrophage signaling by essential messengers produces endogenous H_2_S from cystathionine-β-synthase (CBS) and CSE enzymes. Hydrogen sulfide, a toxic gas that acts by concentration changes, is an efficient gasotransmitter in the modulation of ionic channels and nociceptive receptors. Hydrogen sulfide is pronociceptive in its interaction with Ca_v_3.2 channels and TRPA1 channels and is antinociceptive when it acts on potassium channels and opioid receptors (Domoto et al., 2021; Eken et al., 2022). Hydrogen sulfide acts directly through sulfidation and redox modulation in Ca_v_3.2 with an interaction site on the histidine H191 residue (Peers et al., 2012). Furthermore, it acts indirectly, along with other gasotransmitters such as nitric oxide and carbon monoxide, to modify second-messenger pathways and regulate cytokine and kinase cascades (Li et al., 2020).

This review provides relevant information about hydrogen sulfide modulation on Ca_v_3.2, the main T-type calcium channel subunit participating in neuropathic pain conditions. The remainder of the article is organized as follows: Section 2 briefly describes the classification of T-type calcium channels and the role that other voltage-gated calcium channel subtypes play in neuropathic pain conditions. Then, Section 3 shows recent works depicting the role of the Ca_v_3.2 subtype in neuropathic pain and therapeutic strategies targeting the Ca_v_3.2 channel, such as T-type calcium channel blockers, post-translational regulators of channel cell membrane expression, and redox modulation. As we focus on H_2_S as a modulating molecule of the Ca_v_3.2 channel, some chemical characteristics of H_2_S and its physiological and pathological functions resulting from the complex interaction with some ion channels are explained in Section 4. The most important works demonstrating the CSE/H_2_S/Ca_v_3.2 pathway in the peripheral, central, and visceral nociceptive processes are shown in Section 5. The dual effect of H_2_S/Ca_v_3.2 is discussed in Section 6 according to the literature, and the mechanisms of action proposed to describe this interaction, representing direct and indirect interaction pathways, are given in Section 7. Targeting the CSE/H_2_S/Ca_v_3.2 hydrogen sulfide pathway could be a promising pharmacological strategy for treating neuropathic pain.

## 2 Voltage-gated calcium channels in neuropathic pain transmission

Voltage-gated calcium channels (VGCCs) are essential in nociceptive signal transmission as they participate in various physiological processes, such as neurotransmitter release and regulation of the excitability of DRG primary sensory neurons and dorsal horn neurons (Park and Luo, 2010; Bourinet et al., 2014). VGCCs can be divided into two groups according to the activation voltage threshold: high-voltage-activated (HVA) and low-voltage-activated (LVA) channels. The HVA channels are formed by the channel forming α1 subunit along with auxiliary β, α2δ, and γ subunits. The HVA α1 subunit is codified by the genes *CACANA1S* (α1S), *CACNA1C* (α1C), *CACNA1D* (α1D), and *CACNA1F* (α1S) for the L-type channels and *CACNA1A* (α1A), *CACNA1B* (α1B), and *CACNA1E* (α1E) which form the P/Q-, N-, and R-type channels, respectively. According to this nomenclature, the HVA channels are divided into Ca_v_1 and Ca_v_2 subfamilies, and the Ca_v_1 subfamily comprises the Ca_v_1.1–1.4 subtypes (L-type channels), while the Ca_v_2 subfamily comprises the Ca_v_2.1 (P/Q-type), Ca_v_2.2 (N-type), and Ca_v_2.3 (R-type) channels (Zamponi et al., 2015). The LVA channels, commonly called T-type channels, are formed only by the channel forming α1 subunit codified by the genes *CACNA1G*, *CACNA1H*, and *CACNA1I* for the pore-forming α1G, α1H, and α1I, respectively. The subtypes are named Ca_v_3.1, Ca_v_3.2, and Ca_v_3.3 channels, respectively (Weiss and Zamponi, 2019a).

The L-type HVA channels are composed of four subtypes, namely, Ca_v_1.1–Ca_v_1.4. The L-type can be blocked by benzothiazepines (BZPs) such as diltiazem, dihydropyridines (DHPs) such as nifedipine or nimodipine, and phenylalkylamines (PHEs) such as verapamil. The L-type channels are distributed in skeletal muscle, cardiac myocytes, smooth muscle, endocrine cells, cochlear hair cells, and retinal bipolar cells. The L-type Ca_v_1.1 and Ca_v_1.4 isoforms are expressed mainly in the skeletal muscle and retina, respectively (Zamponi et al., 2015), while the L-type Ca_v_1.2 and Ca_v_1.3 isoforms are distributed primarily in the neurons, including the medium-sized DRG neurons, dorsal horn neuronal cell body, and dendrites. These subtypes are expressed in the postsynaptic membrane to generate neuronal discharges (Li et al., 2019). The Ca_v_1.2 is mainly located in the soma and proximal dendritic ends, and these channels support calcium influx for excitation–transcription coupling underlying the mechanism of nerve injury hyperexcitability in the dorsal horn. Ca_v_1.2 channels mediate calcium transients in persistent pain conditions and are overexpressed in the dorsal horn region. In more detail, Ca_v_1.2 has a dual function as a pore for calcium ion influx and as a transcription factor. The C-fragment of the channel translocates to the nucleus upon channel activation and regulates transcription. It was proven that reducing nuclear calcium signaling decreases the development of chronic inflammatory pain and blocks activation of CREB, but not ERK1/2, in neuropathic pain conditions. In short, nuclear calcium influx by Ca_v_1.2 channels causes alteration of gene expression and long-term changes associated with persistent pathological pain. Ca_v_1.3 is distally located in the somatodendritic tree compartment, playing a role in the expression of plateau potentials and contributing to short-term sensitization to pain (Roca-Lapirot et al., 2018). A mathematical model of the dorsal horn demonstrates that the Ca_v_1.3 channel expression is essential in short-term sensitization in pain transmission, and the Ca_v_1.2 channel contributes to long-term plasticity associated with neuropathic pain (Radwani et al., 2016). The co-administration of opioids and L-type blockers (morphine and nimodipine) represents a therapeutic strategy for analgesic treatment in pain relief (Kumar et al., 2010). Nevertheless, the mechanism is not well-determined since it has been observed that the co-administration of nimodipine and morphine leads to a decreased expression of Ca_v_1.2 and an increase in Ca_v_2.2, and in other studies, it has been demonstrated that there is a decreased level of Ca_v_1.3 but not of the Ca_v_1.2 and Ca_v_2.2 channels (Park and Luo, 2010). Overall, the role of L-type channels in pain therapeutic strategies for neuropathic pain seems unclear.

The P/Q-type HVA channels, also named Ca_v_2.1 channels, are blocked by ω-agatoxin GIVA (isolated from the venom of the funnel web spider *Agelenopsis aperta*). The P/Q-type channels are localized in presynaptic terminals playing a role in the neurotransmitter released (Zamponi et al., 2015). The role of P/Q participation in pain, by its preference central distribution, is mainly related to the familial hemiplegic migraine (FHM) producing headaches and, according to genetic mutations, is associated with defects in R192Q, V714A, T666M, and I1811L residues in the pore-forming α1A subunit (Lorenzon and Beam, 2000; Sekiguchi et al., 2018). The P/Q type in the periaqueductal gray (PAG) region located in the brainstem plays a role in the modulation of the trigeminal nerve. Dysfunctional P/Q channels contribute to migraine pathophysiology in the craniovascular nociception; this was demonstrated in the rat ventrolateral PAG using ω-agatoxin GIVA as a P/Q-type blocker (Knight et al., 2002). It was observed that a lack of 50% of P/Q-type channels reduced allodynia during the initial phase of the chronic constriction injury (CCI) neuropathic pain model. In addition, in a null mutant Ca_v_2.1 pore-forming model, a lack of recovery of the injured nerve was observed, concluding that P/Q-type is fundamental for nerve regeneration (Marinelli et al., 2015). Another study reported that gabapentin, known for its analgesic properties, may inhibit the synaptic transmission effect through P/Q-type channel inhibition in the dorsal horn of the spinal cord in mice (Bayer et al., 2004). Nevertheless, other studies observed no effect of P/Q-type on mechanical allodynia and thermal hyperalgesia using neuropathic pain models, thus making its role in afferent pain signaling unclear (Park and Luo, 2010; Bourinet et al., 2014; Li et al., 2019).

The N-type HVA channels, also named Ca_v_2.2 channels, are blocked by ω-conotoxin GVIA (isolated from the venom of the marine snail *Conus geographus*) (Zamponi et al., 2015). N-type channels are localized in presynaptic nerve terminals in laminae 1 and 2 of the dorsal horn, participating in the release of nociceptive neurotransmitters such as glutamate, substance P, and calcitonin gene-related peptide (CGRP) into spinal interneurons. N-type channels are key targets for inhibition by opioid receptor pathways and play an important role in pain signaling in primary afferent fibers (Zamponi et al., 2009; Bourinet et al., 2014). N-type channels are upregulated in pain conditions after a peripheral nerve injury in the spinal dorsal horn region, and ω-conotoxin MVIIA (derived from *Conus magus*) inhibits the hyperalgesia and allodynia neuropathic conditions (Park and Luo, 2010; Li et al., 2019). The N-type pore-forming α1B subunit splicing variants in exon 37 form exon 37a and exon 37b, with a difference in 14 amino acids located in the carboxyl terminal. A comparison between the exon 37a and exon 37b variants to demonstrate the role of nociceptive effects found a major role of exon 37a in thermal hyperalgesia and sensitivity to morphine molecules (Bourinet et al., 2014). The pregabalin compound acts on Ca_v_α2δ1 to inhibit N-type channels in a spinal nerve ligation (SNL) model (Li et al., 2019; Lanzetti and Di Biase, 2022). The effect of this auxiliary subunit is also studied in the α2δ1 knockout model that inhibits the expression of N-type channels in DRG and dorsal horn neurons on the pain pathway *in vivo* (Nieto-Rostro et al., 2018). These findings conclude that N-type channels are important potential targets for developing novel analgesics. For example, ziconotide, a peptide blocker of N-type channels derived from the ω-conotoxin MVIIA, is administered intrathecally to treat chronic pain in cancer patients (Patel et al., 2018; Gao et al., 2021; Lanzetti and Di Biase, 2022).

The R-type HVA channels, also named Ca_v_2.3 channels, are blocked by the SNX-482 compound. Ca_v_2.3 is expressed in DRG and the reticular nucleus of the thalamus, suggesting participation in peripheral and central nociception. The R-type channels participate in regulating neurotransmitter release and neuronal excitability. The R-type channels have biophysical properties similar to the T-type channels, with the difference in activating a large depolarization stimulation (Zamponi et al., 2015). It was observed that intrathecal formalin-induced pain behavior is attenuated with the SNX-482 blocker, which demonstrated its role in nociception (Terashima et al., 2013). The physalin F compound, isolated from *Physalis acutifolia*, exerts antinociceptive effects through R-type and N-type channels. The physalin F effect was independent of other HVA/LVA channels or G-protein-coupled opioid receptors, and this result was demonstrated using whole-cell and slice electrophysiology on paclitaxel-induced neuropathic pain (Shan et al., 2019). The patch-clamp technique in HEK293 cells proved that L-cysteine increased the R-currents by chelating the trace metal mechanism that tonically inhibits the channel. Furthermore, using the acetic acid visceral pain model with *CACNA1E* knockout, it was found that L-cysteine causes pain responses in wild-type mice and no effect in the *CACNA1E* knockout mice, and these results show that R-type plays a role in visceral pain processing (Ghodsi et al., 2022). Altogether, an upregulation of R-type channels in DRG during neuropathic pain conditions was observed, and these channels also play a role in the pain afferent pathway considering R-type channels a potential target for pain relief (Park and Luo, 2010; Bourinet et al., 2014; Li et al., 2019).

## 3 Ca_v_3.2 T-type calcium channels in neuropathic pain transmission

The T-type LVA channels comprise Ca_v_3.1–Ca_v_3.3 subtypes (Cribbs et al., 1998; Perez-Reyes et al., 1998; Lee J.-H. et al., 1999). The T-type channels can be blocked by divalent and trivalent ions, organic molecules such as mibefradil and ethosuximide, and, recently, TTA-A2 or Z944 nanomolar blockers. The biophysical properties of T-type channels promote regulating the neural excitability processes and oscillations in the membrane potential (Perez-Reyes, 2003; Zamponi et al., 2015). The predominant Ca_v_3 isoform involved in pain processes is the Ca_v_3.2 subtype (Zamponi et al., 2009). Ca_v_3.2 T-type channels are expressed in DRG neurons; in axonal expression, they contribute to the excitability of primary afferent fibers; in the presynaptic terminal of the dorsal horn, they participate in synaptic transmission; and in the midbrain and cortex, they participate in pain processing (Weiss and Zamponi, 2019a; Harding and Zamponi, 2022).

The contribution of Ca_v_3.2 to various pain modalities is well-documented (Cai et al., 2021; Harding and Zamponi, 2022). In this context, to mention some recent findings, it was demonstrated that an increased expression of Ca_v_3.2 channels in somatostatin-positive (SOM^+^) neurons, known as a key population of excitatory interneurons in the spinal dorsal horn, produces thermal hyperalgesia and allodynia. In effect, silencing Ca_v_3.2 channels using knockdown *CACNA1H* in a spared nerve injury reduces neuropathic pain condition (Zhi et al., 2022). In addition, elevated expression and increment of the current density of Ca_v_3.2 channels, but not of Ca_v_3.1 or Ca_v_3.3 channels, located in the superficial spinal dorsal horn contribute to mechanical allodynia in a partial sciatic nerve ligation (PSNL)-induced neuropathic pain model (Feng et al., 2019). The axonal increase in Ca_v_3.2 channels is involved in uninjured afferent nerve fiber sensitization adjacent to spared nerve injury (SNI) and produces mechanical allodynia in neuropathic pain (Chen et al., 2018). The hyperexcitability state of nociceptors in chronic neuropathic pain conditions, induced by spinal cord injury (SCI), is mainly due to the action of T-type channels, representing a ∼60–70% of the inward current of the SCI-nociceptors (Lauzadis et al., 2020). In varicella-zoster virus (VZV) infection, Ca_v_3.2 T-type channels are upregulated in DRG neurons, and the application of (2T/S)-6-PNG inhibitor alleviated mechanical and thermal sensitivity in zoster-associated pain in a mice model (Li et al., 2021). Finally, in the homocysteinemia condition, characterized by an above-normal concentration of homocysteine in the blood, it was found that an increased expression of T-type channels induced mechanical allodynia. This upregulated expression of T-type channels is reached by the protein kinase C (PKC) phosphorylation pathway of three sites, namely, S532A, S1144A, and S2188A, located in the I–II loop, II–III loop, and carboxy-terminal region of the channel, respectively (Gaifullina et al., 2019).

In short, most results show an upregulation of T-currents in neuropathic pain conditions; therefore, the therapeutic strategy focuses on the development of more specific T-type channel blockers. For example, ethosuximide, an antiepileptic drug, has recently been studied for the treatment of chronic pain and pain-related comorbidities, such as anxiety and depression (Kerckhove et al., 2019), as well as the treatment of abdominal pain related to irritable bowel syndrome (IBSET) (Kerckhove et al., 2017). Z944, a more specific T-type channel blocker, potently blocked spinal cord lamina I neurons, reduced excitability in superficial dorsal horn neurons, and reversed mechanical allodynia, demonstrating its use as a therapeutic tool in analgesic treatment (Harding et al., 2021). Z944 also restored cortical synchrony and modified thalamocortical connectivity in a CCI-induced neuropathic pain model by blocking T-type channels (LeBlanc et al., 2016). Novel T-type channels blockers, the prenylated flavonoids, such as (2S)-6-prenylnaringenin (6-PNG), sophoraflavanone G, 2(S)-8-PNG, or synthetic 6-prenylflavanones (6-PFVNs), including (2R/S)-6-PNG and its derivatives, are promising nutraceutical tools to alleviate neuropathic and visceral pain through Ca_v_3.2 T-type channels (Nguyen et al., 2019). The molecular interaction of these nutraceuticals may resemble the molecular interaction of flavonoid derivatives genistein and daidzein over the Ca_v_3.3 channel subtype (Rangel-Galván et al., 2021). Furthermore, an analog of benzimidazolonepiperidine, 5bk, preferentially blocked Ca_v_3.2 channels in a direct way at a low micromolar concentration (IC_50_ = 4.2 µM). The 5bk compound reversed mechanical allodynia in three induced models of HIV sensory neuropathy, paclitaxel-induced neuropathy, and spinal nerve ligation-induced neuropathy in a pathway independent of the interaction of the µ, δ, and κ opioid receptors, representing a non-addictive therapeutic strategy for pain conditions (Cai et al., 2020). The endogenous cannabinoid anandamide targets T-type channels and CB1/CB2 receptors both related to neuropathic pain conditions (Rangel-Galván et al., 2022a; Harding and Zamponi, 2022). Based on these protein targets, a set of synthetic neuromolecular production compounds (NMPs) were developed as antinociceptive promising compounds (Gadotti et al., 2013; Berger et al., 2014; Rangel-Galván et al., 2022b). In this context, camphene and alpha-bisabolol, terpenes derived from *Cannabis* plants, show a partial inhibition of 25.4% and 29.3% on Ca_v_3.2 channels in tsA-201 cells and in DRG neurons, respectively. The IC_50_ values were 4.5 and 7.7 µM for alpha-bisabolol and camphene, respectively. These molecules reduced thermal hyperalgesia in mice (Gadotti et al., 2021).

**FIGURE 2:**
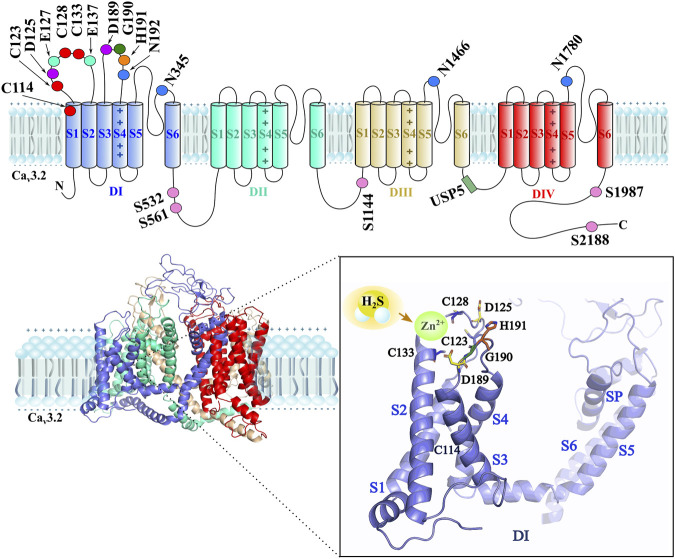
The *upper part* shows a schematic 2D representation of the main interaction sites of H^2^S/Ca^v^3.2 are located in the first domain of the channel (Domain I-DI), such as the extracellular cysteines C114, C123, C128, and C133 (red), the aspartic acid D125 and D189 (purple), the glutamic acid E127 and E137 (green cyan), the glycine G190 (dark green), and the histidine H191 (orange) residue as main interaction sites by H_2_S. The asparagines N192, N345, N1466, and N1780 (blue) residues are glycosylation sites. The phosphorylation sites are the serines S532, S561, S1144, S1987, and S2188 (pink) residues. The ubiquitination site (forest green) by the USP5 enzyme is located in the DIII–DIV intracellular loop. The *lower part* shows a 3D structure of the Ca_v_3.2 channel, where the close-up of the DI indicates the main interaction sites by H_2_S on the Ca_v_3.2 channel isoform, including zinc in the redox site. The D189–G190–H191 motif and C133, C123, and C128 cysteine residues, along with D125 and D189 aspartic acid residues, stabilize Zn^2+^ when H_2_S interacts in the redox modulation.

Another strategy for treating the upregulation of T-type channels in pathological conditions is to target the post-translational modifications such as N-linked glycosylation, phosphorylation, and ubiquitination that participate in the trafficking process that regulates the expression of T-type channels in the cell membrane. The glycosylation modifies asparagine N192 and N1466 residues, adding extracellular sugar groups to increase Ca_v_3 expression (Harding and Zamponi, 2022). It has been demonstrated that blocking the glycosylation process by de-glycosylation enzymes, such as neuraminidase (NEU) and PNGase-F (PNG), reduces the Ca_v_3.2 effect *in vitro* by reducing T-current densities in whole-cell recordings and *in vivo* by reducing diabetic hyperalgesia in type 1 diabetes condition (Joksimovic et al., 2020). Phosphorylation of T-type channels increases its expression in the cell membrane. The cyclin-dependent kinase 5 (CDK5) upregulates Ca_v_3.2 expression in DRG and spinal dorsal horn neurons, producing mechanical allodynia induced by spinal nerve ligation (SNL) in rats. Mutagenesis shows that S561 and S1987 residues are the important regulation sites (Gomez et al., 2020). Ubiquitination also regulates the T-type channel expression. The Ca_v_3.2/ubiquitin-specific protease 5 (USP5) interaction shows a way of inhibiting the overexpression of T-type channels in pathological conditions like pain conditions (Bourinet et al., 2016; Harding and Zamponi, 2022). Intracellular lysine residues in the domain II–IV linker region in the Ca_v_3.2 T-type channel are important for USP5 interaction (García-Caballero et al., 2014). The bortezomib compound increased the protein levels of Ca_v_3.2 in DRG and induced peripheral neuropathy through the upregulation of USP5 that inhibits proteasomal degradation of Ca_v_3.2 in mice (Tomita et al., 2020). In this matter, the endofacial structure of T-type channels is a target of therapeutic strategy since it presents more variability among other channel isoforms in comparison with the high homology present in the exofacial side of the T-type channels (Weiss and Zamponi, 2019b). Figure 2 schematically shows the main post-translational regulation sites on the Ca_v_3.2 channel.

Finally, it is worth mentioning that redox modulation of T-type channels is a recommendable pharmacological strategy. It has been proposed that regulating the channel function is an improved physiological strategy rather than blocking it through antagonists (Todorovic and Jevtovic-Todorovic, 2014). The important role of the increased activity of Ca_v_3.2 T-type channels has been considered due to the action of the reducing agent H_2_S, which participates in visceral, inflammatory, and neuropathic pain. This H_2_S/Ca_v_3.2 pathway establishes a new investigation guide to develop novel therapeutic strategies for neuropathic pain relief (Sekiguchi et al., 2018).

## 4 Hydrogen sulfide and ion channel interactions

Hydrogen sulfide is an endogenous gasotransmitter along with carbon monoxide (CO) and nitric oxide (NO). All these gaseous molecules are of importance in regulating physiological processes. Hydrogen sulfide is formed endogenously from L-cysteine by four routes that involve the enzymes: a) cystathionine-γ-lyase (CSE), b) cystathionine-β-synthase (CBS), c) cysteine aminotransferase (CAT) with 3-mercaptopyruvate sulfurtransferase (3MST), and d) cysteine lyase (CL). CBS and CSE are cytosolic; CBS and 3MST are distributed mainly in the central nervous system (CNS), whereas CSE is located in the peripheral nervous system. 3MST is zinc-dependent and, along with CAT, has both mitochondrial and cytosolic distributions (Li et al., 2011; Mir and Maurya, 2018b). Another endogenous pathway of H_2_S production from persulfide and polysulfide compounds involves enzymatic-independent mechanisms like glycolysis, phosphogluconate, and intracellular reductants (Kolluru et al., 2017). H_2_S is a weak acid (pKa = 6.76 at 37°C), lipophilic, and colorless with a rotten egg odor and has a boiling temperature of −60.7°C. It dissociates in an aqueous solution to form hydrogen ions (H^+^) and hydrosulfide anions (HS^−^) to form 2H^+^ and S^2−^ ions later. Under physiological conditions, H_2_S exists primarily in the form of monoanion SH^−^ (82%), deprotonated H_2_S (18%), and dianion S^2−^ (<0.1%). The hydrosulfide anion can be oxidated to form sulfite (SO_3_^2−^), sulfate (SO_4_^2−^), thiosulfate (S_2_O_3_^2−^), and other polysulfide species. H_2_S is a powerful reducing agent at a concentration >1,000 ppm and may cause adverse effects in CNS (Li et al., 2011; Powell et al., 2017; Mir and Maurya, 2018a; Liu et al., 2022). The main routes at which H_2_S exerts biological functions include metal center interactions, reactive oxygen species (ROS), reactive nitrogen species (RNS) scavenging, and S-persulfidation (Powell et al., 2017). On ion channels and receptors, H_2_S is regulated through protein sulfhydration, channel redox modulation, and indirect effects by interaction with other gasotransmitters like carbon monoxide and nitric oxide, as well as secondary pathway regulation (Peers et al., 2012). S-persulfidation, commonly known as S-sulfhydration, is the process of conversion of a thiol (R-SH) group into a perthiol (R-SSH) (Powell et al., 2017). The physiological level of H_2_S in brain tissue is 50–160 µM and that in human and rat serum is 50–100 µM, as measured spectrophotometrically. Recent estimates measure that H_2_S free concentration in mouse brain and liver homogenates is at an approximate value of 15 nM (Li et al., 2011).

The general physiological function includes cell differentiation, development vasodilation, and immune responses (Liu et al., 2022). In the peripheral nervous system, H_2_S modulates cardiac, vascular, gastrointestinal, urogenital, respiratory, and endocrine functions. In the central nervous system, H_2_S is involved in hippocampal long-term potentiation (LTP), producing both neurotoxicity and neuroprotection. The therapeutic potential involves pain, neurodegeneration, cardiovascular, inflammatory, infectious, and neuropathological disease approaches (Li et al., 2011; Wallace and Wang, 2015; Liu et al., 2022). The interaction of H_2_S with ion channels includes the ability to interact with the vascular smooth muscle K_ATP_, K^+^ channels (K_v_2.1), big conductance Ca^2+^-sensitive K^+^ (BK_Ca_), L-type Ca^2+^ channels, T-type Ca^2+^ channels, intracellular chloride channels (Cl^−^), transient receptor potential vanilloid (TRPV) channels, and transient receptor potential ankyrin-1 (TRPA1) (Li et al., 2011; Peers et al., 2012; Fukami and Kawabata, 2015). In addition, H_2_S also interacts with the sodium/calcium exchangers (NCX), β-adrenergic receptors, and N-methyl-D-aspartate receptors (NMDA) in different cells (Zhang et al., 2015). H_2_S interacts with transcription factors such as nuclear factor κB (NF-κB) and tumor necrosis factor α (TNF-α), and with kinases such as the mitogen-activated protein kinase (MAPK), the extracellular signal-regulated kinase (ERK) 1/2, and the protein kinase C (PKC) (Li et al., 2011).

Several uncertainties exist concerning the H_2_S mechanism of action to play roles in physiological or pathophysiological conditions. The overall effect of H_2_S indeed depends on the local concentration. Nevertheless, it is challenging to measure exact H_2_S real-time intracellular concentration, and as reviewed previously, the value has changed from micromolar to nanomolar concentrations. Another factor to consider is to monitor other H_2_S species such as HS^−^ and sulfide S^2−^ to give us an idea of the total concentration, given that the species concentrations of H_2_S exist primarily as HS^−^ monoanion. It is worth noting that H_2_S is a highly diffusible gas and, when formed, is likely to be rapidly sequestered or catabolized, having a time of action that could be another variable to consider in the H_2_S mechanism of action description. As we mentioned previously, among the variety of targets, H_2_S regulates many types of ion channels. For example, there is evidence that H_2_S activation of K_ATP_ contributes to the protection of myocardial ischemia/reperfusion injury and the neuroprotection against glutamate-induced toxicity. Nevertheless, there is evidence that H_2_S increased intracellular calcium concentrations through L-type channels. This calcium concentration leads to glutamate release and subsequent neurotoxicity, involving the NMDA receptors in this process (Li et al., 2011; Peers et al., 2012). These contradictory results show us the complexity of the H_2_S gasotransmitter, where the mechanism of action to play roles in physiological or pathophysiological scenarios involves the concentration, the time of action, and the signaling pathways.

## 5 CSE/H_2_S/Ca_v_3.2 neuropathic pain pathway

To investigate the role of H_2_S in peripheral nociceptive processes, intraplantar (i.pl.) administration of NaHS (H_2_S donor) and L-cysteine (endogenous precursor of H_2_S) was performed in a model to produce hyperalgesia in rats. The presence of Fos expression was observed in the spinal dorsal horn. The use of the oxidizing agent, 5,5′-dithiobis-(2-nitrobenzoic acid) (DTNB), and the Ca_v_3.2 channel blockers (ethosuximide and mibefradil) reversed the hyperalgesia produced by H_2_S. Furthermore, the use of DL-propargylglycine (PPG) and β-cyanoalanine (BCA) compounds, irreversible and reversible inhibitors of the enzyme cystathionine-γ-lyase (CSE), respectively, partially inhibited hyperalgesia induced by i.pl. lipopolysaccharide (Kawabata et al., 2007). To determine the role of the Ca_v_3.2 channel in the central and peripheral nociceptive processing systems by the action of H_2_S, intrathecal (i.t) and intraplantar (i.pl.) administration of NaHS (H_2_S donor) was performed in a model. The NaHS donor sensitizes the Ca_v_3.2 channels, leading to hyperalgesia. Mibefradil, zinc chloride inhibitors, and the antisense oligodeoxynucleotides (AS-ODNs) that caused the silencing of the Ca_v_3.2 channel in the dorsal root ganglia (DRG) and spinal cord alleviated hyperalgesia induced by i.pl. and i.t NaHS (Maeda et al., 2009). To prove the participation of the enzyme cystathionine-γ-lyase (CSE) in the H_2_S–Ca_v_3.2 pathway of neuropathic pain, irreversible and reversible inhibitors of this enzyme, the DL-propargylglycine (PPG) and β-cyanoalanine (BCA) compounds, were used, respectively. The i.pl. administration of PPG and BCA reversed the neuropathic hyperalgesia and allodynia induced by i.pl. NaHS. The neuropathic model was induced by L5 spinal nerve injury (L5SNC) in rats. Upregulation of the Ca_v_3.2 channel was observed; the participation of this channel was evaluated using mibefradil blocker and RNA interference (RNAi) to silence the Ca_v_3.2 channel, and both actions attenuated the neuropathic hyperalgesia in the L5SNC rat model (Takahashi et al., 2010).

The involvement of CSE in the H_2_S–Ca_v_3.2 pathway of neuropathic pain was confirmed in a mechanical hyperalgesia model obtained by a repeated systemic administration of paclitaxel, an anti-cancer drug. The i.pl. administration of PPG and BCA reversed the paclitaxel-evoked neuropathy. The role of the Ca_v_3.2 was studied by the knockdown of the channel by intrathecal administration of AS-ODNs and the NNC 55-0396 and mibefradil blockers in HEK293 cells. No upregulation of Ca_v_3.2 channels was detected in DRG, spinal cord, and peripheral tissues (Okubo et al., 2011). In addition to the H_2_S–Ca_v_3.2 pathway, the activation of the transient receptor potential ankyrin-1 (TRPA1) channel leads to mechanical hyperalgesia and allodynia induced by i.pl. administration of NaHS in mice. The Ca_v_3.2 role was studied by the use of NNC 55-0396, mibefradil, ascorbic acid, and zinc blockers, and AP18 blocker was used for the TRPA1 channel. Furthermore, silencing Ca_v_3.2 and TRPA1 proteins were achieved by AS-ODN methodology. The inhibition of both proteins in sensory neurons reverses mechanical hyperalgesia and allodynia in mice (Okubo et al., 2012). The function of Ca_v_3.2 channels is enhanced by endogenous H_2_S synthesized by CSE. This affirmation was demonstrated using PPG as a CSE inhibitor, which decreased the calcium currents through the Ca_v_3.2 channels transfected in HEK293 cells. The exogenous H_2_S enhanced the Ca_v_3.2 channel when the endogenous H_2_S production was inhibited by PPG. Among the H_2_S donors, Na_2_S is more potent than NaHS *in vitro* and *in vivo*, increasing Ca_v_3.2 currents in the range of 0.1–0.3 mM compared to 1.5 mM, respectively. In addition, the i.pl. administration of Na_2_S at 10 pmol/paw in comparison with 100 pmol/paw of NaHS produced mechanical allodynia/hyperalgesia which was reversed by pretreatment with the NNC 55-0396 Cav3.2 blocker (Sekiguchi et al., 2014).

The CSE/H_2_S/Ca_v_3.2 pathway is involved in visceral nociception, including colonic, pancreatic, and bladder pain (Kawabata and Matsunami, 2012). In colonic pain, abdominal allodynia/hyperalgesia induced by intracolonic NaHS/capsaicin involves secondary regulation via the increase in phosphorylation of extracellular signal-regulated protein kinase (ERK) in the spinal dorsal horn. The allodynia/hyperalgesia induced by intracolonic NaHS was alleviated by the Ca_v_3.2 mibefradil blocker (Matsunami et al., 2009). In pancreatic pain, the pancreatitis-related abdominal allodynia/hyperalgesia was induced by repeated doses of caerulein in mice and the injection of NaHS/capsaicin in the pancreatic duct. The injection of NaHS/capsaicin produced the delayed expression of Fos protein and phosphorylation of ERK in the superficial layers of the spinal dorsal horn, and those actions were reversed by the Ca_v_3.2 mibefradil blocker. An upregulation of CSE was also observed during the development of pancreatitis (Nishimura et al., 2009; Kawabata and Matsunami, 2012). Cystitis-related bladder pain induced by the i.pl. administration of cyclophosphamide in mice confirmed the CSE/H_2_S/Ca_v_3.2 pathway using PPG as a CSE inhibitor, mibefradil and NNC 55-0396 as blockers of the Ca_v_3.2 channel, and AS-ODNs to silence the channel. In addition, an increase in phosphorylation of ERK was observed in the superficial layer of the L6 spinal cord after the NaHS administration (Matsunami et al., 2012).

Although the T-type channel blockers used to demonstrate the nociceptive role of Ca_v_3.2 channels are not specific to this channel, for example, mibefradil has been seen to target other ion channels like Na^+^ (McNulty et al., 2006) and K^+^ channels (Hong et al., 2012), it is observed that new generation of T-type channel blockers show the same effects. In effect, the i.pl. and intracolonic (i.col.) administration of Na_2_S produced mechanical allodynia and visceral nociceptive behavior, respectively. The administration of a recent Ca_v_3.2 blocker TTA-A2 alleviated mechanical allodynia in the Ca_v_3.2 knockout C57BL/6 mice (Matsui et al., 2019). The ascorbic acid reduces somatic and visceral pain induced by the i.pl. and i.col. administration of NaHS in a model of paclitaxel-evoked neuropathy in GNL/SMP30-KO mice (Tsubota et al., 2019). Due to the involvement of the CSE/H_2_S/Ca_v_3.2 pathway in neuropathic and visceral pain, the therapeutic targets are focused on these elements to develop future pharmacologic compounds.

## 6 Dual effect of H_2_S on ionic channel modulations

The dual effect of H_2_S, NO, and CO is known in all these gas molecules cataloged as toxic pollutants. In general, the exposure level of 10 ppm H_2_S has no metabolic effects and concentrations higher than 30 ppm cause symptoms such as headache, nausea, and vomiting, while 150–250 ppm concentration exposures cause respiratory tract irritation and pulmonary edema. For CO, the exposure level of 0.5–5 ppm is acceptable, 100 ppm exposure level causes headaches, dizziness, and nausea, and 5,000–6,000 ppm concentration levels produce loss of consciousness. At the same time, NO at 400 ppm exposure level produces loss of consciousness (Li et al., 2009). From these molecule gases, at a physiological concentration level, a dual excitatory/inhibitory effect of NO has been observed for a long time, acting by concentration on different receptors and neural ion channels in the context of pain. Indeed, applying SIN-1 (NO donor) at different doses, it is found that at low concentrations (0.1–2.0 µg/10 µL) reduced tactile allodynia, at medium concentrations (5 or 100 µg/10 µL), it has no effect; and at high concentrations (10 and 20 µg/10 µL), it increased the mechanical allodynia induced by chronic ligature of sciatic nerve in rats (Sousa and Prado, 2001).

A dual effect of H_2_S on T-type calcium channels is described at the molecular level using calcium imaging and patch-clamp recording techniques. In a range of 10 μM–1 mM of NaHS, inhibition of ∼30% in the Ca_v_3.2 channels in HEK293 cells, ∼18.6% in DRG neurons, ∼25% in NG108-15 cells, and ∼18–20% in HL-1 cells was observed. According to the authors, low concentrations are physiologically relevant to determine the effect of H_2_S/Ca_v_3.2 interaction, and the mechanism proposed is that H_2_S inhibits Ca_v_3.2 via increasing the affinity of Zn^2+^ to the channel in the H191 site (Elies et al., 2014; 2016). Intracellular calcium signals measured by the calcium imaging (Fura-2/AM) technique show that NaHS (10 µM) decreased the resting intracellular calcium concentration [(Ca^2+^)_i_] through T-type channels. The T-type blocker nickel (100 µM) prevents decreasing [Ca^2+^]_i_ induced by H_2_S (Avanzato et al., 2014). Another study uses DL-propargylglycine PPG, a CSE inhibitor, at 0.95 and 5 mM concentrations for 10 min, showing reduced T-currents. A reduction of the enzyme that produces H_2_S (CSE), in turn, reduces the T-currents. The authors hypothesize that the endogenous form H_2_S acts tonically to enhance T-currents (Sekiguchi et al., 2014). Nonetheless, a half-time decay concentration of H_2_S of 6.2 ± 0.1 min has been measured (García-Bereguiaín et al., 2008), and it would be necessary to propose a mechanism that may maintain a continuous concentration of H_2_S to produce an enhancement in T-type calcium channels (Table 1).

**TABLE 1:** H_2_S concentration effect on various ionic calcium channels.

Channel	Location	H_2_S effect	Concentration	H_2_S donor	Technique	Reference
T-type	HEK293 cells	Inhibition	10 μM–1 mM	NaHS	Patch-clamp (whole-cell)	Elies et al. (2014)
T-type	Rat cardiomyocytes (H9c2)	Inhibition	10 µM	NaHS	Calcium imaging (Fura-2/AM)	Avanzato et al. (2014)
T-type	HEK293 cells	Augmentation	3 mM	NaHS	Patch-clamp (whole-cell)	Elies et al. (2014)
T-type	HEK293 cells	Augmentation	100 µM	Na_2_S	Patch-clamp (whole-cell)	Matsui et al. (2019)
T-type	DRG	Augmentation	1.5 mM	NaHS	Patch-clamp (whole-cell)	Matsunami et al. (2009)
T-type	NG108-15 cells	Augmentation	1.5 mM	NaHS	Patch-clamp (whole-cell)	Kawabata et al. (2007)
T-type	Astrocytes (hippocampal slices)	Augmentation	200 µM	NaHS	Calcium imaging (Green-1/AM ester)	Nagai et al. (2004)
T-type	Rat GH3 pituitary tumor cells	Augmentation	200 µM	NaHS	Calcium imaging (Fura-2/AM)	Yong et al. (2010)
L-type	Mouse pancreatic β-cells	Inhibition	65.4 µM	NaHS	Patch-clamp (whole-cell)	Tang et al. (2013)
L-type	Rat cardiomyocytes	Inhibition	100–1,000 µM	NaHS	Patch-clamp (whole-cell)	Zhang et al. (2012)
L-type	Rat cardiomyocytes (H9c2)	Inhibition	10 µM	NaHS	Calcium imaging (Fura-2/AM)	Avanzato et al. (2014)
L-type	Rat cardiomyocytes	Inhibition	25–400 µM	NaHS	Patch-clamp (whole-cell)	Sun et al. (2008)
					Calcium imaging (Fluo-3/AM)	
L-type	Astrocytes (hippocampal slices)	Augmentation	200 µM	NaHS	Calcium imaging (Green-1/AM ester)	Nagai et al. (2004)
L-type	Cerebellar granule neurons (CGNs)	Augmentation	100 µM pulses each 10 min (50–120 µM)	NaHS	Calcium imaging (Fura-2/AM ester)	García-Beregiaín et al. (2008)
L-type	Rat GH3 pituitary tumor cells	Augmentation	200 µM	NaHS	Calcium imaging (Fura-2/AM)	Yong et al. (2010)
BK	hiPSC-MSC	Inhibition	100 µM	NaHS	Patch-clamp (whole-cell)	Zhao et al. (2013)
BK	Mouse aortic endothelial cells (MAECs)	Augmentation	100 µM	Na_2_S	Patch-clamp (whole-cell)	Chai et al. (2015)
BK	Rat GH3 pituitary tumor cells	Augmentation	300 µM	NaHS	Patch-clamp (whole-cell)	Sitdikova et al. (2010)
N-type	Astrocytes (slices)	Augmentation	200 µM	NaHS	Calcium imaging (Green-1/AM ester)	Nagai et al. (2004)
TRP	Astrocytes (slices)	Augmentation	200 µM	NaHS	Calcium imaging (Green-1/AM ester)	Nagai et al. (2004)
NMDA	Rat GH3 pituitary tumor cells	Augmentation	200 µM	NaHS	Calcium imaging (Fura-2/AM)	Yong et al. (2010)

T-current augmentation was observed using 3 mM of NaHS on Ca_v_3.2 channels in HEK293 cells and 10 mM of NaHS in DRG neurons (Elies et al., 2014). Matsunami et al. (2009) usedwhole-cell patch-clamp recordings on DRG neurons of small diameter (<30 µm) and showed an increment by 1.5 mM of NaHS but not in 0.5 or 3 mM concentration of H_2_S donor. The same effect was observed in whole-cell recordings in NG108-15 cells using dithiothreitol (DTT), a reducing agent, 5,5′-dithiobis-(2-nitrobenzoic acid) (DTNB), an oxidizing agent, and NaHS as an H_2_S donor, suggesting that the effect of 0.1 mM DTNB abolished the T-type calcium current and that 1 mM of DTT and 1.5 mM NaHS enhanced the T-type calcium current (Kawabata et al., 2007). Matsui et al. (2019) conducteda patch-clamp study using Na_2_S as an H_2_S donor at 100 μM concentration and showed that Ba^2+^ currents increased by almost double the control amplitude in Ca_v_3.2 channels expressed in HEK293 cells. Overall, T-type calcium channels had an inhibitory effect at a low concentration of H_2_S in the order of 10 µM and an augmentation of T-current at > 100 µM H_2_S concentration.

The dual effect is also observed in L-type, another voltage-gated calcium channel from the HVA family. In the pancreatic β-cells, where L-type plays a role in regulating insulin secretion, H_2_S induced an inhibition of the steady-state calcium current by NaHS (IC_50_ = 65.4 µM) by 31.3% and 100 µM of the latanoprost analog ACS 67 (slow-releasing H_2_S donor) by 18% (Tang et al., 2013). In the excitation/contraction coupling in cardiomyocytes, increasing concentrations of NaHS at 100, 200, 500, and 1,000 μM/L reduced the amplitude of the peak of L-current by 85.1%, 79.5%, 74.4%, and 62.0%, respectively (Zhang et al., 2012). In this same system, intracellular calcium signals measured by fluorometric live cell imaging showed that NaHS (10 µM) decreased the resting [Ca^2+^]_i_, and the nifedipine (10 µM) L-type blocker prevented the [Ca^2+^]_i_ decrease induced by H_2_S (Avanzato et al., 2014). This inhibitory effect of L-type by H_2_S was observed using patch-clamp (whole-cell) recordings, where NaHS (100 µM) reduced the peak of L-current in isolated cardiomyocytes. Changing the concentration of NaHS at 25, 50, 100, 200, and 400 µM determines a Kd of 87.4 µM for NaHS and 84 µM for H_2_S. The administration of H_2_S has shown a cardioprotective effect in various disease models (Sun et al., 2008).

On the other hand, the administration of NaHS increased [Ca^2+^]_i_ in astrocytes in culture producing calcium waves and in hippocampal slices producing an augmentation effect in various ionic channels. Nifedipine, flurazine, ω-conotoxin blockers for L-type, T-type, and N-type calcium channels, respectively, in addition to La^3+^, Gd^3+^, Mg^2+^, MDL-12, and 330 A blockers of the TRP family eliminate the effect of H_2_S (Nagai et al., 2004). H_2_S raises cytosolic calcium in cerebellar granule neurons (CGNs) by activation of L-type channels. The pathophysiological conditions were simulated by the administration of 100 µM NaHS pulses every 10 min to maintain a final H_2_S concentration range of 50–120 µM. It was observed that H_2_S half-time decay concentration is 6.2 ± 0.1 min. The exposure of CNG cells to 200–300 µM H_2_S concentration for an hour produced the rise of [Ca^2+^]_i_ to generate a neurotoxic environment and induced the death of nearly 50% of neurons. The cell viability was determined with MTT [3-(4,5-dimethylthiazol-2-yl)-2,5-diphenyl tetrazolium bromide] assay. Nifedipine and nimodipine (IC_50_ < 1 µM) prevented the effect of H_2_S (García-Bereguiaín et al., 2008). In the SH-SY5Y cell line, the effect of H_2_S induced an increase in [Ca^2+^]_i_ after the administration of 200 µM NaHS; this effect was attenuated in L-type (∼37%), T-type (∼55%), and N-methyl-D-aspartate (NMDA) (∼40%) using the blockers verapamil, mibefradil, and MK-801, respectively (Yong et al., 2010). Finally, the BK channel also showed an increase (Sitdikova et al., 2010; Chai et al., 2015) and inhibition (Zhao et al., 2013) effect by the action of the H_2_S in different systems.

Table 1 summarizes information corresponding to the type of ion channel, location, effect after interacting with H_2_S, concentration, H_2_S donor, technique with which the effect on the channel was evaluated, and the reference. This table allows us to see more clearly if there is a dependence on the concentration of H_2_S on an inhibitory or increasing effect on a particular ion channel. In this review, we emphasize the role of H_2_S in T-type calcium channels. Even though, in the T-type calcium channels, the H_2_S appears to have a dual effect base on the concentration, in other ionic channels, it is not so determinant because the increased or decreased effect of H_2_S is seen in a micromolar range administration of NaHS donor, for example, in L-type and BK channels. It is possible that the location and the set of ionic channels and receptors expressed in the cell, in addition to the action of secondary pathways, determine the final effect of H_2_S modulation.

## 7 Proposed mechanism of action of hydrogen sulfide over the Ca_v_3.2 channels

### 7.1 Direct mechanism

The direct interaction of hydrogen sulfide with ion channels mainly involves two mechanisms of action: protein sulfhydration and channel redox modulation (Peers et al., 2012). The S-sulfhydration mechanism includes the replacement of cysteine thiol (Cys-SH group) by the -SSH group by the action of hydrogen sulfide (Kimura, 2015). Although the sulfhydration processes have been observed in other ion channels like K_ATP_ (Fukami and Kawabata, 2015; Kimura, 2015), TRPA1 (Ogawa et al., 2012), and L-type calcium channel (Zhang et al., 2012), the sulfhydration mechanism in T-type calcium channels is not the most important mechanism. In fact, it has been observed that the application of cysteine-modifying agent N-ethylmaleimide (NEM) and the disulfide bond-modifying methodology UV light pulses did not alter the effect of the reducing compounds dithiothreitol (DTT) and L-cysteine to enhance the T-currents. The NEM agent significantly attenuated the inhibition by DTNB, meaning that the effects of oxidizing agents but not reducing agents depend on the modification of conserved cysteine residues. The metal chelators diethylenetriaminepenta-acetic acid (DTPA) and N, N, N, N-tetrakis (2-pyridylmethyl) ethylenediamine (TPEN) can reproduce the effect of the L-cysteine, suggesting that the Ca_v_3.2 channel is also modified by metal chelation (Nelson et al., 2005; Evans and Todorovic, 2015).

The Ca_v_3.2 channels have a high-affinity blockade site by divalent (Zn^2+^, Cu^2+^, and Ni^2+^) and trivalent ions (Y^3+^, La^3+^, and Gd^3+^) (Mlinar and Enyeart, 1993) known as extracellular metal binding site (EMBS). Among these metals, Ni^2+^ has been traditionally used as a T-type channel blocker; the Ca_v_3.2 channel is more sensitive to this blocker than the other two subunits (IC_50_ = 13 µM for Ca_v_3.2 compared with an IC_50_ = 250 µM and IC_50_ = 216 µM for Ca_v_3.1 and Ca_v_3.3, respectively) (Lee et al., 1999a; Sun et al., 2007). Ni^2+^ and Zn^2+^ block preferentially the Ca_v_3.2 subunit, producing an allosteric effect on channel gating instead of a direct blockade over the permeation pathway (Shuba, 2014). The Zn^2+^ inhibits Ca_v_3.2 with an IC_50_ = 0.8 µM and IC_50_ = 80 µM for Ca_v_3.1 and IC_50_ ∼ 160 µM for Ca_v_3.3 (Lory and Chemin, 2007; Traboulsie et al., 2007). From these two ions, Zn^2+^ is more physiologically relevant due to its participation in different pathways in *in vivo* mechanisms; for example, it acts as a neurotransmitter in glutamatergic neurons regulating membrane receptors (Mathie et al., 2006).

The EMBS includes the H191 residue that forms a motif that includes the residues Asp189–Gly190–H191 located in the IS3–IS4 and Asp in IS2 (Kang et al., 2010). Using the reducing agent L-cysteine and endogenous metal chelators of Zn^2+^, it has been demonstrated that the molecular basis mechanism of peripheral C-type dorsal root ganglion nociceptors involves the H191. This important key residue is present in the Ca_v_3.2 isoform but not in the Ca_v_3.1 and Ca_v_3.3 channels (Nelson et al., 2007b). The Ca_v_3.2 mutant H191Q and Ca_v_3.1 mutant Q191H demonstrate the importance of the H191 residue for Ca_v_3.2 channel modulation by H_2_S (Elies et al., 2014). The ascorbate, or L-ascorbic acid or vitamin C, is an oxidizing agent inhibiting the Ca_v_3.2 channels at a nanomolar concentration (IC_50_ = 10 nM) (Nelson et al., 2007a). On the other hand, the α-lipoic acid LA (1,2-dithiolane-3-pentanoic acid), an endogenous oxidizing compound, also has a molecular mechanism of analgesic action through the Ca_v_3.2 channels. However, unlike other compounds, LA acts similarly on Ca_v_3.1 and Ca_v_3.2. Electrophysiological recordings and mutagenic studies demonstrated that the mechanism occurs via oxidation of specific extracellular thiol groups of cysteine (C934A, C123A, C128A, and C133A) in repeats I and II instead of the H191 of the Ca_v_3.2 channel to inhibit the Ca_v_3.2 current. Furthermore, *in vivo* studies demonstrate the LA decreased sensitivity to noxious thermal and mechanical stimuli in mice, which was not observed in Ca_v_3.2 knockout mice (Woo et al., 2009). As previously described in detail, the effect of oxidizing and reducing agents involving H_2_S/Ca_v_3.2 interaction showed that the reducing agents L-cysteine and DTT compound interact with Ca_v_3.2 to enhance the current, leading to nociceptor sensitization and neuropathic condition (Nelson et al., 2005; Kawabata et al., 2007; Fukami and Kawabata, 2015). On the other hand, the oxidizing agents, ascorbic acid (vitamin C), zinc chloride, and DTNB compound, led to the inhibition of Ca_v_3.2 channels to alleviate neuropathic effects (Kawabata et al., 2007; Maeda et al., 2009; Okubo et al., 2012; Elies et al., 2014; Tsubota et al., 2019).

From recent studies, it is hypothesized, according to experimental mutagenic evidence, using MTSES (methanethiosulfonate) and NEM (N-ethylmaleimide) as modifying cysteine agents, that the extracellular cysteines C114, C123, C128, and C133 can modify the local redox environment when suffering oxidative modification producing an increased sensitivity of zinc affinity and allosteric changes to the metal binding site of the Ca_v_3.2, favoring channel inhibition. Nevertheless, determining the most probable cysteine oxidate structures, such as cysteine sulfinic (Cys-SO_2_H) or cysteine sulfonic (Cys-SO_3_H), requires considering additional studies (Huang et al., 2020). From this group of cysteine, the least sensitive was C123, and observing the presence of a disulfide bond in the Ca_v_3.1 channels subtype, the authors predict a disulfide bond formation in C123–C939 residues. Altogether, the evidence suggests a major channel redox modulation by the action of H_2_S gasotransmitter involving mainly the H191 residue. Nevertheless, the cysteine groups C114, C123, C128, and C133 can participate in the mechanism of action of H_2_S over Ca_v_3.2 channels. Figure 2 shows the proposed mechanism of H_2_S/Ca_v_3.2 interaction through redox modulation. According to Todorovic and Jevtovic-Todorovic., 2014, the His191 could suffer metal-catalyzed oxidation (MCO); this reaction is exemplified with ascorbic acid (Todorovic and Jevtovic-Todorovic, 2014). In another mechanism, extracellular cysteines in the IS1–IS2 loop suffer oxidative modification increasing Ca_v_3.2 inhibition by liberating zinc of the H191 site (Huang et al., 2020). A contrast mechanism describes a stabilization of zinc in the H191 site by the action of H_2_S (Elies et al., 2016).

### 7.2 Indirect mechanism

The indirect mechanism involves interactions with other gasotransmitters, such as carbon monoxide (CO) and nitric oxide (NO), the microglia-related cytokines, and kinase regulation. T-type calcium channels in DRG are modulated by NO gasotransmitter by the S-nitrosothiol (SNO) mechanism on cysteines located in repeats I and II on the extracellular loop of the channel producing an inhibition in native and recombinant Ca_v_3.2 T-channels (Lee et al., 2013). Furthermore, using CORM-2 as a CO donor, CO is reported to inhibit native and recombinant T-type channels with an IC_50_ ~ 3 μM for all three subtypes. The inhibition mechanism may be mediated by a redox-sensitive site only for the Ca_v_3.2 channel (Boycott et al., 2013). It is important to mention that NO and CO participate as neural modulators in interweaving signaling pathways with the H_2_S ion channel modulation pathway. Each of these gasotransmitters can influence the expression and activity of the enzymes that generate their biosynthesis, a phenomenon known as “crosstalk.” Simultaneously, generating NO/CO/H_2_S in pathological conditions overregulates the production of the iNOS/HO-2/CSE enzymes that synthesize them (Li et al., 2009). On this interweaving, it is observed that H_2_S can regulate the NO/cGMP/PKG pathway producing an antinociceptive effect through µ-opioid receptors (Li et al., 2020), and through the PKG, could enhance the Ca_v_3.2 channels to produce a pronociceptive effect (Iftinca and Zamponi, 2009). The elevation of cGMP and activation of PKG by H_2_S can involve the gasotransmitter CO (Wang, 2012). Therefore, there exists a complex interaction between the gasotransmitters and the Ca_v_3.2 channel in the neuropathic pain context (Peers et al., 2012; Gamper and Ooi, 2015).

The neuropathic pain condition is mediated by microglia activation in the spinal cord. For example, the inflammatory cytokine IL-6 is an important mediator in developing neuropathic pain conditions. The effect of H_2_S is the inhibition of interleukin-6 (IL-6) in the spinal cord (Kida et al., 2015). On the other hand, it has been demonstrated that IL-6 upregulates Ca_v_3.2 channels in DRG neurons in the development of neuropathic pain. The mechanism is that elevated IL-6 binds to a soluble molecule sIL-6R forming the IL-6/sIL-6R complex that activates the receptor gp130 (producing homodimerization of the receptor) that triggers the JAK signaling cascade producing functional upregulation of T-type channels (Liu et al., 2019). H_2_S has an anti-inflammatory effect, alleviating mechanical allodynia and thermal hyperalgesia, by reducing nuclear factor-kappa B (NF-κb) expression and the excessive inflammatory cytokines tumor necrosis factor (TNF-α), interleukins IL-1β and IL-6, and high mobility group box (HMGB-1) in the spinal cord after peripheral nerve injury via the nuclear factor erythroid-2 (Nrf2)/hemeoxygenase-1 (HO-1) pathway in the microglial cells (Chen et al., 2019). It is found that elevated levels of interleukin-1β (IL-1β) promote interaction between USP5 and Ca_v_3.2 channels such that it may participate in maintaining a chronic pain state (Stemkowski et al., 2017). The interaction of TNF-α/Ca_v_3.2 has been studied in the context of axon growth by promoting Ca^2+^ influx through these channels (Kisiswa et al., 2017), which means that interaction in neuropathic pain can occur, promoting a pronociceptive effect. There is also evidence that T-type calcium channels can activate the Nrf2/HO-1 pathway in cisplatin-induced auditory cell damage, using flunarizine as a T-type antagonist, and decrease proinflammatory cytokines (TNF-α, IL-6, IL-1β, etc.) (So et al., 2008).

**FIGURE 3:**
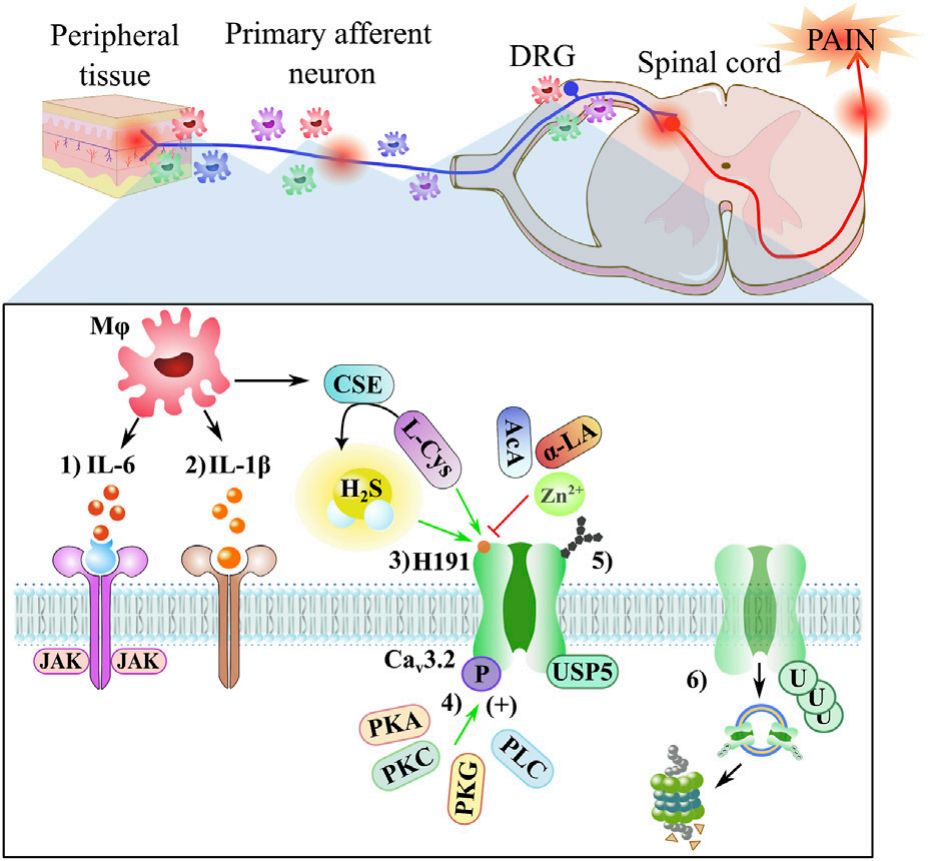
Neuropathic pain involves neuro-immune crosstalk. The peripheral nerve macrophages/spinal microglia (Mφ) accumulate after a nerve injury around nociceptors and DRG, inducing a variety of mediators, such as IL-6, IL-1β, and CSE enzyme. A close-up shows a schematic representation of some elements of the indirect regulation via Ca_v_3.2 channels: 1) IL-6 (red) binds to sIL-6R (blue) and induces homodimerization of gp130 (purple) to activate the JAK signaling cascade that triggers upregulation of the Ca_v_3.2 channel in DRG neurons; 2) the IL-1β (orange) binds to IL1R (brown), producing a second messenger pathway; 3) the reducing agents, such as L-cysteine (L-Cys) and H_2_S molecule, augment T-currents, while the oxidizing agents, such as ascorbic acid (AcA), α-Lipoic acid (α-LA), and Zn^2+^ ion, inhibit T-currents, both processes occur through the H191 residue; 4) the Ca_v_3.2 channel can be phosphorylated through kinases, such as PKA, PKC, PKG, and PLC enzymes; 5) the Ca_v_3.2 channel can be regulated through the glycosylation site; and 6) the expression of the Ca_v_3.2 channel is regulated by USP5, a deubiquitinating enzyme that protects the channel from proteasomal degradation.

The slow-releasing H_2_S donors A-ITC and P-ITC inhibited behaviors associated with neuropathic pain conditions by blocking extracellular signal-regulated kinase 1/2 (ERK1/2) phosphorylation (Cabarga et al., 2020). In cystitis-related bladder pain, NaH increased the function of ERK kinase, while the NNC 55-0396, a T-type blocker, inhibited that effect (Matsunami et al., 2012). A more detailed description of H_2_S interaction with transcriptor factors and kinases is reviewed by Li et al., 2011. The ERK1/2 participation in H_2_S modulation through T-type calcium channels remains to be determined (Peers et al., 2012). Macrophages can upregulate CSE and increase the production of H_2_S, mediating pathological pain through Ca_v_3.2 T-type and TRPA1 channels, and the activation of a series of receptors increased the activation of protein kinase A (PKA), protein kinase C (PKC), phospholipase C (PLC), mitogen-activated protein kinase (MAPK), which in turn enhances the activity of Ca_v_3.2 T-type (Sharma et al., 2023) and TRPA1 channels to produce pronociceptive effects (Domoto et al., 2021). Figure 3 shows some indirect interaction that regulates Ca_v_3.2 channel expression, including the microglia-related cytokines, kinase regulation, and the post-translational modifications such as the N-linked glycosylation, phosphorylation, and ubiquitination processes.

## 8 Conclusion

Neuropathic pain conditions involve disorders in the signaling pathways of the somatosensory system produced by primary injuries and collateral damage due to some chronic diseases. The L-type HVA calcium channels in neuropathic pain plays an indirect role and could participate through the α2δ1 modulation subtype by the action of gabapentin and pregabalin or through the interaction with opioid receptors. For P/Q-type HVA, it is clear that its involvement in migraine is associated with headaches, but in neuropathic conditions, there are contradictory results, so more investigation is needed to establish the mechanism of these HVA subtypes. For N-type and R-type, it is clear that they are promising targets in the context of neuropathic pain treatments. Nevertheless, we focus on the T-type LVA calcium channels by their importance in this pathology; from the three subunits that comprise this family of channels, the Ca_v_3.2 subunit has more relevant participation through its biophysical properties and distribution covering the central and peripheral nervous system areas. Indeed, Ca_v_3.2 is located in the primary afferent fibers, the dorsal root ganglia neurons, the dorsal horn neurons, the midbrain, and the cortex, which covers all the sensory pathways. It is well-documented that the upregulation of Ca_v_3.2 channels or an increase in the intracellular concentration of calcium through these channels is related to mechanical allodynia and hyperalgesia in a variety of systems, including visceral pain. The therapeutic strategies include the design of more selective blocking molecules for these channels at nanomolar concentrations, such as TTA-A2, TTA-P2, or Z944 T-type blockers. A line of investigation focused on molecules that can be subtype selective, and this strategy considered the distribution of the channel subtypes, with Ca_v_3.2 having a greater preference for the periphery and Ca_v_3.1 subtype for the central areas. A set of new molecules are derivatives of natural sources, such as flavonoids, which are nutraceutical tools for treatment. The upregulation of Ca_v_3.2 can also be regulated through post-translational modifications like glycosylation, phosphorylation, and ubiquitination. In addition, a final strategy considers the redox modulation, which is directed to the Ca_v_3.2 subtype because it acts on the H191 residue present in this subtype but not in Ca_v_3.1 or Ca_v_3.3 channels. One of the molecules that modulate this site (H191) is hydrogen sulfide H_2_S, which was previously considered a toxic gas, but a series of studies have helped to classify it as a gasotransmitter, along with NO and CO, which, at low concentrations, have physiological and therapeutic importance. H_2_S, in neuropathic pain conditions, can interact with a set of ionic channels and receptors, and recent studies find dual participation, which can be antinociceptive through the interaction with potassium channels and pronociceptive through Ca_v_3.2 channels. A series of studies have demonstrated the importance of the CSE/H_2_S/Ca_v_3.2 pathway in neuropathic pain conditions. Reviewing with more detail the H_2_S/Ca_v_3.2 interaction, we found that the action of H_2_S over Ca_v_3.2 depends on the concentration of H_2_S and could interact in a direct or indirect form. The direct redox modulation involves the participation of Zn^2+^ and the oxidation state of a group of cysteines surrounding the H191 residue. In an indirect mechanism, H_2_S can regulate the Ca_v_3.2 through cytokines and kinases, not to mention the interaction with other gasotransmitters. Overall, the mechanism is complicated, but understanding the CSE/H_2_S/Ca_v_3.2 pathway represents a promising therapeutic strategy for treating neuropathic pain conditions.
